# Optimization, Characteristics, and Functions of Alkaline Phosphatase From *Escherichia coli*

**DOI:** 10.3389/fmicb.2021.761189

**Published:** 2022-02-21

**Authors:** Yachao Dong, Yandong Xia, Jie Yin, Diao Zhou, Yidan Sang, Sufeng Yan, Qingshu Liu, Yaqi Li, Leli Wang, Ying Zhao, Cang Chen, Qiuyun Huang, Ying Wang, Muhammad Nazeer Abbasi, Huansheng Yang, Chuni Wang, Jianzhong Li, Qiang Tu, Jia Yin

**Affiliations:** ^1^Hunan Provincial Key Laboratory of Animal Intestinal Function and Regulation, Hunan International Joint Laboratory of Animal Intestinal Ecology and Health, The National and Local Joint Engineering Laboratory of Animal Peptide Drug Development, College of Life Sciences, Hunan Normal University, Changsha, China; ^2^CAS Key Laboratory of Quantitative Engineering Biology, Shenzhen Institute of Synthetic Biology, Shenzhen Institutes of Advanced Technology, Chinese Academy of Sciences (CAS), Shenzhen, China; ^3^College of Life Sciences and Technology, Central South University of Forestry and Technology, Changsha, China; ^4^Hunan Institute of Microbiology, Changsha, China

**Keywords:** alkaline phosphatase, inflammatory factor, IPEC-J2 cell line, immunity, disease

## Abstract

Weaning of piglets could increase the risk of infecting with Gram-negative pathogens, which can further bring about a wide array of virulence factors including the endotoxin lipopolysaccharide (LPS). It is in common practice that the use of antibiotics has been restricted in animal husbandry. Alkaline phosphatase (AKP) plays an important role in the detoxification and anti-inflammatory effects of LPS. This study investigated the protective effects of AKP on intestinal epithelial cells during inflammation. Site-directed mutagenesis was performed to modulate the AKP activity. The enzyme activity tests showed that the activity of the DelSigD_153_G-D_330_N mutants in *B. subtilis* was nearly 1,600 times higher than that of the wild-type AKP. In this study, an *in vitro* LPS-induced inflammation model using IPEC-J2 cells was established. The mRNA expression of interleukin-(IL-) 6, IL-8, and tumor necrosis factor-α (TNF-α) were extremely significantly downregulated, and that of ASC amino acid transporter 2 (ASCT-2), zonula occludens protein-1 (ZO-1), and occludin-3 (CLDN-3) were significantly upregulated by the DelSigD_153_G-D_330_N mutant compared with LPS treatment. This concludes the anti-inflammatory role of AKP on epithelial membrane, and we are hopeful that this research could achieve a sustainable development for the pig industry.

## Introduction

Weaning is a critical stage of mammalian postpartum growth and intestinal development. The early stages of weaning are usually associated with decreased performance (Pié et al., 2004: 641-7) and increased incidence of intestinal diseases and diarrhea in animals (Fairbrother et al., 2005: 17-39), which would subsequently result in increasing the risk of infecting with Gram-negative pathogens, such as *E. coli*, *S. typhimurium*, etc. Therefore, it is necessary to prevent gut disorder and diseases by maintaining proper barrier function. Previously, farmers were gravitated to add different antibiotics into the feeds to prevent or reduce pathogenic infections. However, unregulated use of antibiotics has led to several problems, such as antibiotic resistance, adverse drug reactions, and accumulation of drug residues in animal products. Considering the mentioned issues, it is compelling to develop effective alternatives to replace antibiotics in animal feeds and to promote high-quality development of animal husbandry. Under existing commercial conditions, antibiotic alternatives include probiotics, organic acids, auxins, prebiotics, synbiotics, enzymes, antimicrobial peptides, hyperimmune eggs antibodies, clay, and metal (Gadde et al., 2017: 26-45).

The Gram-negative pathogens produce virulence factors including the endotoxin lipopolysaccharide (LPS). Functional LPS binds to Toll-like receptors (TLR-4) on immune cells through the main regulatory nuclear factor-κB (NF-κB) pathway, consequently activating the secretion of proinflammatory cytokines (Goldberg et al., 2008: 3551-6), such as tumor necrosis factor-α (TNF-α), interleukin- (IL-)1, IL-6, IL-8, and IL-12 (O’Neill et al., 2013: 453-60). The increase in proinflammatory factors leads to the destruction and localization of tight junction protein levels, thereby increasing the passage of intestinal contents into the systemic circulation (Liu et al., 2016: 1009-17). The main functions of AKP include improvement of the intestinal barrier function (Liu et al., 2016: 1009-17), prevention of bacterial translocation (Estaki et al., 2014: 15650-6), and detoxification and anti-inflammatory effects of local or systemic intestinal LPS (Bilski et al., 2017: 9074601). AKP dephosphorylates the lipid A portion of LPS and no longer causes TLR-4 stimulation. AKP may even act as TLR-4 receptor antagonist in preventing its binding to the receptor (Molnár et al., 2012: 3254-9), which is essential for maintaining the normal intestinal homeostasis and inhibiting its transport across the intestinal mucosal barrier (Molnár et al., 2012: 3254-9).

Tight junctions are a kind of junction polyprotein complexes, which are located at the tip of the outer membrane of epithelial cells and serve as a barrier preventing solute diffusion across the intercellular space. The breakdown of tight junctions increases paracellular permeability, which allows the migration of harmful substances, such as pathogens and endotoxins, leading to tissue damage and inflammation. Previous studies have shown that the upregulation of zonula occludens protein-1 (ZO-1) and occludins (CLDN) can inhibit the increase in intestinal permeability caused by tight junction disruption in weaned piglets. Therefore, maintaining appropriate expression levels of CLDN and ZO-1 are widely considered to be an effective target for the treatment of intestinal diseases (Omonijo et al., 2019: 615-24).

Previous studies have concluded that AKP is effective in reducing the risk of Gram-negative bacterial infection, blocking the secretion of LPS-activated proinflammatory cytokines. In addition, AKP is a regulator of intestinal mucosal permeability and appears to work through improving tight junction protein levels and localization (Liu et al., 2016: 1009-17). The cells were incubated with LPS detoxification by AKP, and mRNA transcriptional levels of inflammatory factors were monitored; the mRNA levels of ASC amino acid transporter 2 (ASCT-2), CLDN-3, and ZO-1 also were tracked. The proinflammatory cytokines, such as IL-6, IL-8, and TNF-α, were related to the occurrence of immune function or pathological damage and significantly decreased, indicating that AKP can effectively alleviate the pathological injury of immune function to achieve anti-inflammation (Omonijo et al., 2019: 615-24). CLDN-3 and ZO-1 gene expression play an important role in maintaining barrier function and are exceptionally upregulated, revealing that AKP can effectively improve intestinal barrier function, possibly by preserving tight junction protein formation and integrity (Liu et al., 2016: 1009-17). ASCT-2 gene expression mainly absorbs neutral amino acids, such as glutamine, alanine, serine, and cysteine, and is greatly upregulated, providing important nutrients for cell metabolism (Xu et al., 2020: 111376).

*E. coli* has the ability to grow rapidly on cheap substrates, but it produces virulence factors, including the endotoxin LPS, and therefore cannot be used as feed additives. In recent years, the use of Gram-positive *B. subtilis* strains to produce recombinant proteins has been widely a concern by the academia and industry (Schallmey et al., 2004: 1; Liu et al., 2013: 6113-27). Previous studies have shown that *B. subtilis* can easily absorb extracellular genetic material as nutrients or expand the diversity of genotypes (Chen et al., 2005: 1456-60). In addition, *B. subtilis* has superior ability to exocrine proteins and other metabolites and without producing endotoxins, which makes it an ideal expression host for industrial and pharmaceutical applications (Fu et al., 2008: 287-92; Demain and Vaishnav, 2009: 297-306; Caspers et al., 2010: 1877-85; Cai et al., 2017: 70).

In this study, we have used homologous recombination coupled with site-directed mutagenesis techniques to construct a stable plasmid with high expression activity of the AKP in *B. subtilis*. By improving the activity of AKP to effectively prevent and control livestock and poultry diseases, reducing dependence on antibiotics, and promoting the healthy and harmonious development of the livestock and poultry industry, these meaningful findings of AKP would provide a certain reference for sustainable development of the pig industry.

## Materials and Methods

### Cell Line and Culture Conditions

The IPEC-J2 cell line is derived from porcine IPEC intestinal epithelial cells and was purchased from Otwo Biotech^1^. The cells were grown and stored in a complete medium containing DMEM (89% high sugar, Hyclone) supplemented with 10% special-grade fetal bovine serum (phage-free low endotoxin, TIANHANG^2^) and 1% antibiotics (penicillin and streptomycin, Gibco). The AKP recombinant strain was cultured overnight at 37°C aerobically, and they were passed at least twice before the experiment. The culture supernatant was prepared by passing the supernatant through a sterile filter with a 0.22-μm pore size (Hippo’s). After filtering with a sterile syringe filter, the protein concentration was measured accurately through the Take3™ multivolume plate (Thermo Scientific). The protein sample was normalized to 2 μg/ml and pretreated with 10 μg/ml of LPS at 37°C for 1 h, then added into cells and incubated for 24 h.

### Strains and Plasmids

The strains used in this study are listed in Supplementary Table 1. GB05-dir was used for DNA recombineering (Fu et al., 2012: 440-6). It was incubated in LB aerobic medium (10 g of tryptone, 5 g of yeast extract, 5 g of NaCl per liter, and the solid medium is added with 1.4% agar) in a Thermo Mixer (Eppendorf) at 37°C, 900 rpm. Wild-type *B. subtilis* was used as host for heterologous expression of recombinant AKP. They were inoculated with GM I and then transferred into GM II medium for the plasmid transformation. The specific ingredients are as shown in Supplementary Table 2. The plasmid pSC101-BAD-ETgA-tet has the pSC101 origin, L-arabinose inducible promoter, and tetracycline resistance gene. The plasmid pP43NMK-APN-Cm-Km contains a broad-host pP43NMK origin and conveys kanamycin and chloramphenicol resistance. Appropriate antibiotics were added at the following concentrations when necessary: kanamycin and chloramphenicol were used for *E. coli* strains at 10 μg/ml, tetracycline was used for *E. coli* strains at 5 μg/ml, and the concentration of L-arabinose used for induction was 1.4 mg/ml.

### Screening of Alkaline Phosphatase Gene

We used the NCBI online tool^3^ to search the open reading frame (initiation codon--termination codon) of AKP genes from different species. The signal peptide cleavage site of the obtained AKP was analyzed by online analysis software^4^. The porcine AKP genes have introns and are available through artificial gene synthesis [Bioengineering (Shanghai) Co., Ltd.^5^ ]. *E. coli* and *B. subtilis* AKP gene are obtained through PCR. The open reading frames of four AKP genes, including *phoA* (GenBank accession No. CP037857.2), *phoE* (GenBank accession No. CP053102.1), *ncdF* (GenBank accession No. CP052842.1), and *apn* are collected (GenBank accession No. AH012163.2). These expression plasmids containing AKP genes from different species were constructed by the Red/ET DNA recombineering technology. The AKP genes were placed behind the tetracycline inducible promoters (P_*tet*_), and kanamycin and chloramphenicol resistance genes were also added into the constructs. The culture was induced with 0.2 μg/μl of tetracycline and incubated at 37°C for 4 h. The AKP activity was detected with the AKP kit (Nanjing Jiancheng Institute of Biological Engineering^6^).

### Construction of *Escherichia coli–Bacillus subtilis* Shuttle Vector (pP43NMK)

The plasmid pP43NMK-*APN*-Cm-Km was digested with PstI to expose the homology arms and to recover the 6,845-bp fragment as a linear vector. The AKP gene *phoA*, *phoE*, and *nudF* were amplified from the plasmids pGB-Cm-ccdA-Ptet-*phoA*, pGB-Cm-ccdA-Ptet-*phoE*, and pGB-Cm-ccdA-Ptet-*nudF.* The oligonucleotides P43-*phoA*-5/P43-*phoA*-3, P43-*phoE*-5/P43-*phoE*-3, and P43-*nudF*-5/P43-*nudF*-3 were used for PCR. The oligonucleotide sequences are listed in Supplementary Table 3. Two hundred nanograms of PCR products and 200 ng of pP43NMK linear vector were co-electroporated into the L-arabinose-induced GB05-dir, and the recombinants were selected by kanamycin on LB agar plates at 37°C overnight and identified by restriction analysis and sequencing.

Alkaline phosphatase expression plasmids are modified and optimized to achieve high levels of production. First, the signal peptide of AKP gene was deleted, and DelSig*phoA* was prepared from the plasmid pGB-Cm-ccdA-Ptet-*phoA* by designing the fragment P43-DelSig-5/P43-*phoA*-3. Based on the characteristics of bioinformatics AKP, directed mutagenicity was carried out on the active sites of AKP based on the removal of signal peptides. Previous studies found that AKP mutant strains D_101_A (Yun et al., 2013: 533-8), D_153_G (Dealwis et al., 1995: 865-71), E_322_Y (Hou and Cui, 2012: 229-46), K_328_Y (Wojciechowski et al., 2002: 903-11), and D_330_N (Le Du et al., 2002: 941-53) could improve AKP activity. A series of mutant expression plasmids were constructed to select mutant strains with high AKP activity. In this study, the toxin–antitoxin system (mazE–mazF system) was introduced into the expression plasmids to prevent loss of expression plasmids, the bacterial solution at a concentration of 10^–5^ was incubated for generations, and the stability of the plasmid was tested by counting the colony number.

### Simulation Protocol

The initial structures of PhoA enzyme, DelSig*phoA*, and DelSigD_153_G-D_330_N were generated by *I-tasser* (Yang and Zhang, 2015: W174-81). Disodium phenyl phosphate was docked to these three proteins by *Autodock* (Morris et al., 2009: 2785-91), respectively. Molecular dynamic simulation was used to inspect the stability of each complex. All the calculations were performed by *Amber20* (Case et al., 2015: 1-884). Hydrogen atoms of protein were added by the Tleap module based on the Amber ff14SB force filed (Ponder and Case, 2003: 27-85). The systems were soaked in TIP3P (Jorgensen et al., 1983: 926-35) water box with a 10-Å buffer. The sodium ions were added to neutralize the whole systems. The structures were then minimized by 4,000 steps of the steepest descent followed by 1,000 steps of conjugate-gradient descent. There was 80 ps of molecular dynamics simulations in the NPT ensemble during which the temperature was increased from 0 to 300 K. After this initial relaxation, each system was simulated for 20 ps in the NPT ensemble. All the systems were then simulated in the NVT ensemble in 100 ns with a time step of 2 fs. The trajectories obtained from the NVT ensemble were used for subsequent data analysis. For all three complexes, the root-mean-square-deviations (RMSDs) of all heavy atoms were calculated with the reference to the initial conformation.

### Precipitation of Extracellular Proteins Including Alkaline Phosphatase

The strain of the DleSig*phoA* and DleSigD_153_G-D_330_N were inoculated on LB agar plates containing kanamycin and cultured overnight at 37°C. The AKP in the supernatant was precipitated by acid, acetone, ethanol, and salt. (1) Acid, 2% HCl, was slowly added into the supernatant so that the final pH of the mixed solution was reached to 4, 4.5, 5, 5.5, and 6 for 30 min. (2) Acetone, the supernatant, was precipitated with 50% final concentration of acetone solution for 30 min. (3) Ethanol, the supernatant, was precipitated with 50% final concentration of ethanol solution for 30 min. (4) Salt, the supernatant, was precipitated with 50% NaCl saturation solution for 30 min. They were centrifuged at 4°C, 9,000 rpm/min for 30 min to collect precipitates and then suspended in 100 μl of LB liquid medium to detect the AKP activity.

### Quantitative Real-Time PCR

Total RNA was isolated from the cells by following the instructions of the manufacturer. The RNA concentration and OD_260_/OD_280_ value were accurately measured by using Take3™ multivolume plate. Reverse transcription of 2 μg of total RNA into cDNA used Prime Script™ RT reagent Kit with gDNA Eraser (Perfect Real Time) (RR047A, Takara) according to the instructions of the manufacturer. TB Green Premix Ex Taq™ II (RR820A, Takara) was chosen for real-time PCR reaction to analyze the transcription levels of proinflammatory factors in Applied Biosystems Real Time PCR System (Thermo Fisher Scientific). The target genes were IL-6, IL-8, and TNF-α, as well as ASCT 2, CLDN 3, and ZO-1. The reference genes β-actin and cyclophilin A (Cyc A) were being studied. The primers used for qPCR are listed in Supplementary Table 3. Two microliter of cDNAs were directly added in the PCR reaction mixture with a final volume of 20 μl, containing 10 μl of TB Green Premix Ex Taq II (Tli RNaseH Plus) (2 ×), 0.8 μl of PCR Primer (10 μM), and 0.4 μl of ROX Reference Dye or Dye II (50 ×). The amount of the reverse transcription reaction solution should not exceed 10% of the total volume of the PCR reaction solution. The thermal curve of all reactions was performed at 95°C for 3 min, then 95°C for 5 s for 40 cycles, and at 60°C of 34 s. The amplification curve and melting curve of real-time PCR were confirmed after the reaction was completed, and a standard curve for PCR quantification was prepared to ensure the specificity of the reaction. The amount of PCR product was calculated by Delta Delta C(T) method.

## Results

### Screening and Activity Optimization of the Alkaline Phosphatase

**FIGURE 1:**
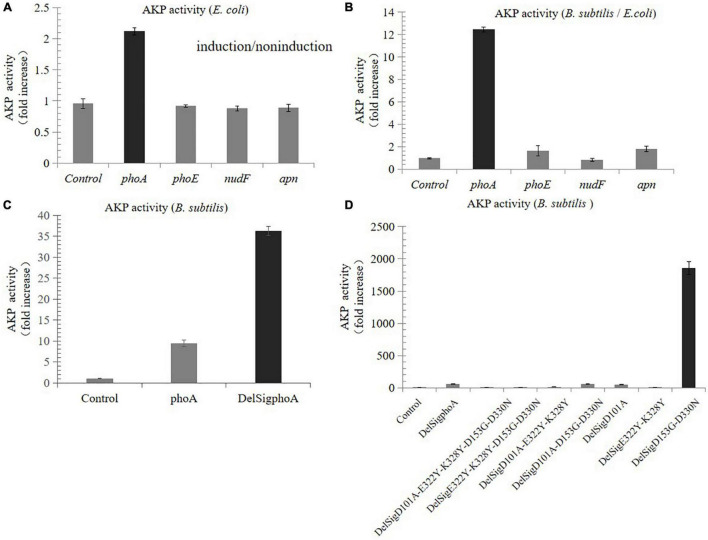
Screening and improving enzyme activity of AKP. **(A)** Fold increase in the AKP activity in *Escherichia coli* by tetracycline inducible promoter. **(B)** Fold increase in the AKP activity in *Bacillus subtilis* compared with *E. coli*. **(C)** Fold increase in the AKP activity without signal peptide and with signal peptide in *B. subtilis*. **(D)** Fold increase in the AKP activity in *B. subtilis* by point mutation. Control is empty vector in *E. coli* or *B. subtilis*. Error bars, SD; *n* = 3.

Compared with the non-induced control, the AKP activity of the recombinants derived from *E. coli* was nearly increased by two times, while that from porcine and *B. subtilis* was not increased (Figure 1A). The results of enzyme activity assay showed that the activity derived from the *phoA* gene of *E. coli* was still the highest in *B. subtilis*, which was 12.5 times higher than that in *E. coli* (Figure 1B). In order to further improve the enzymatic activity, the signal peptide was also deleted by Red/ET recombineering. The results revealed that the activity of DelSig*phoA* was significantly increased, and it was four times higher than that of the wild-type *phoA* (Figure 1C). A series of mutants were constructed using Red/ET recombinant engineering. Results revealed that the activity of the DelSigD_153_G-D_330_N mutants were the highest, which was 32 times higher than that of the DelSig*phoA* (Figure 1D).

The RMSDs of the generated structures of PhoA enzyme, DelSig*phoA*, and DelSigD_153_G-D_330_N were calculated. The RMSD between PhoA and DelSig*phoA* is 0.52 Å, 0.60 Å between PhoA and DelSigD_153_G-D_330_N, and 0.57 Å between DelSig*phoA* and DelSigD_153_G-D_330_N. The three complex structures of the docking results are shown in Supplementary Figures 1A–C and are the binding pockets of the PhoA–, DelSig*phoA*–, and DelSigD_153_G-D_330_N–ligand complexes, respectively. It shows that the ligand strongly interacted with LYS-328 and ARG-166 of both DelSig*phoA* and DelSigD_153_G-D_330_N. Therefore, these LYS and ARG may be the key residues for the enzymatic reaction. As can be seen, the side chain of ASP-153 in PhoA enzyme and DelSig*phoA* points to the center of the binding pocket. Mutation of this residue into GLY may eliminate steric hindrance and, thus, improve enzyme activity, which has been confirmed in this study (Figure 1D). During the molecular dynamic simulations, the RMSD of each complex with the reference of the docking conformation was also calculated. The RMSDs of PhoA, DelSig*phoA*, and DelSigD_153_G-D_330_N were maintained at 3.0–4.5, 2.5–3.0, and 3.0 Å, respectively. The results showed that the mutations had no effect on the structural stability. Therefore, site-directed mutagenesis increases the activity of the enzyme without affecting the structural stability.

It was also observed that these plasmids were unstable and rapidly lost from the cells, which were growing without antibiotics. In this study, the toxin–antitoxin system (mazE-mazF system) (Zhang et al., 2006: e71) was introduced into the expression plasmid by Red/ET recombineering. The MazE was the antidote to the toxin MazF of *E. coli*. Colony-forming unit counting was also performed to test the plasmid stability in the strains, and the results revealed that the stability of DelSigD_153_G-D_330_N mutants has been significantly improved (Supplementary Figure 2).

### Precipitation of Extracellular Proteins Including Alkaline Phosphatase

**FIGURE 2:**
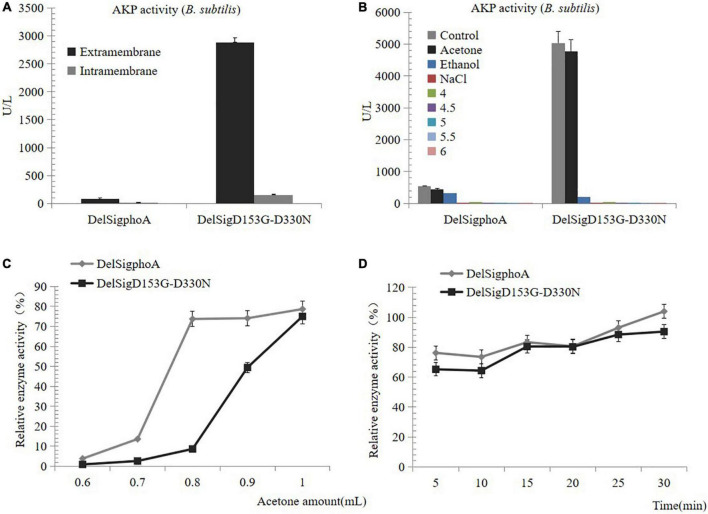
Precipitation of AKP. **(A)** Distribution of AKP in fermentation broth. **(B)** Precipitation of AKP with acid, acetone, ethanol, and salt precipitation. **(C)** The effect of amount of acetone on AKP activity. **(D)** The effect of incubation time on AKP activity under acetone concentration (v/v) of 50%. Error bars, SD; *n* = 3.

To purify the AKP, we first detected the enzyme activity of the supernatant and the centrifugal pellets. The result revealed that the enzymatic activity of the supernatant accounted for the majority (Figure 2A). The AKP enzyme protein was precipitated by acid, acetone, ethanol, and salt precipitation. No enzyme activity was detected at pH 4, 4.5, 5, 5.5, 6, or with salt precipitation treatment. Conversely, a certain activity was observed by the treatment with acetone or ethanol, and the highest precipitation was observed with acetone treatment. Therefore, it can be inferred that acetone was the best reagent for AKP enzyme precipitation (Figure 2B). The collection of high concentrations of AKP requires mass culture. The supernatant of DelSig*phoA* and DelSigD_153_G-D_330_N was treated with different proportions of acetone. We found that within a certain range, the greater the dosage of acetone, the AKP enzyme activity would be the higher (Figure 2C). The acetone treatment time was optimized to 50% (v/v) of the acetone concentration. The results revealed that within a certain range, the longer the treatment time, the higher the activity of AKP, and it reached the peak at 30 min (Figure 2D).

### Thermal Stability of Alkaline Phosphatase

**FIGURE 3:**
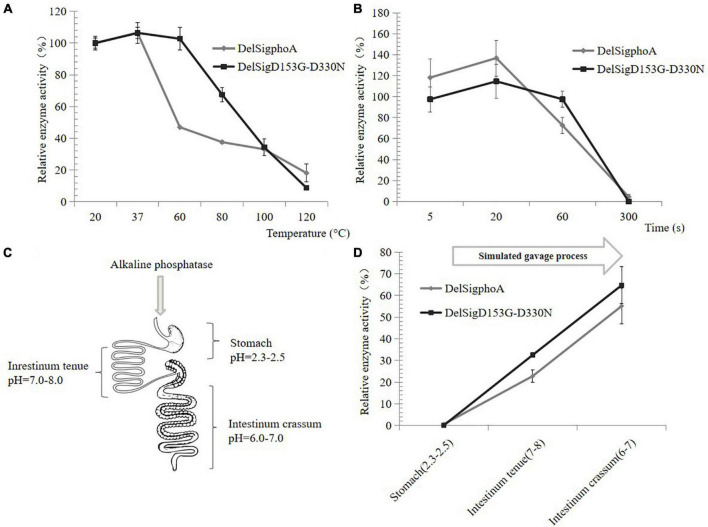
Heat, acid, and alkali stability of AKP. **(A)** Heat stability of AKP on different temperatures. **(B)** The effect of incubation time at 120°C on AKP activity. Control is the vector. **(C)** Schematic diagram of piglet gastrointestinal after absorption of the AKP. **(D)** The effect of AKP on acid and alkali environment for simulating gavage process. Error bars, SD; *n* = 3.

The thermal stability of AKP was also tested at high temperature. When the temperature was at 60°C, the relative activity of DelSig*phoA* was significantly reduced and only retaining 40% of its activity. The enzyme activity of the DelSigD_153_G-D_330_N did not change significantly. The enzyme activity of both constructs still retained 20% with increasing temperature up to 120°C (Figure 3A). Normal spray drying was retained for 5 s at high temperature. The activity of the construct was detected by shortening the treatment time at 120°C. After 1 min of treatment, the enzyme activity of DelSig*phoA* decreased by 60%, while that of DelsigD_153_G-D_330_N decreased by only 20%. The results showed that the thermal stability of DelSigD_153_G-D_330_N was superior to that of DelSig*phoA*, and it still maintains higher enzymatic activity even after 1 min of treatment at 120°C (Figure 3B). As can be seen, the AKP appears to be dried at a relatively high temperature and retained high enzyme activity.

### Dynamic Simulation for Measuring the Alkaline Phosphatase Activity *in vitro*

The AKP would pass through the digestive system of the piglet with different pH values (Figure 3C); thus, an *in vitro* measurement system of the AKP activity was established. The AKP activity of DelSig*phoA* and DelSigD_153_G-D_330_N was tested in different pH ranges and revealed that no activity was detected for the DelSig*phoA* or DelSigD_153_G-D_330_N under acidic conditions (stomach: pH = 2.3–2.5). After adjusting the pH value simulated to intestinum tenue (pH = 7–8), the AKP activity was restored to 20% and 30% for DelSig*phoA* and DelSigD_153_G-D_330_N, respectively. Subsequently, the pH value was changed into 6–7 simulated to intestinum crissum. The AKP activity was recovered to 55% and 65% for DelSig*phoA* and DelSigD_153_G-D_330_N, respectively (Figure 3D). The results suggested that the AKP was stable with a significant loss of enzyme activity under strong acidic conditions. Meanwhile, its activity was resumed to a certain extent from acidic to alkaline conditions.

### The Effect of Alkaline Phosphatase on IPEC-J2 Cells

**FIGURE 4:**
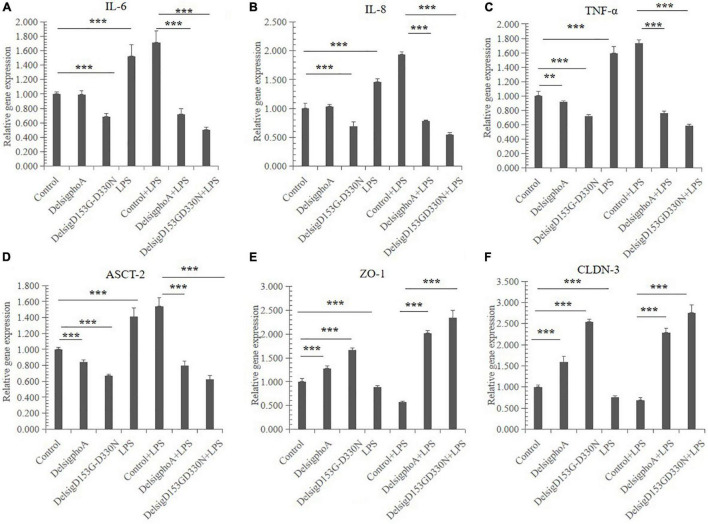
The effect of AKP on relative mRNA abundances in LPS-induced IPEC-J2 cells. **(A)** The effect of AKP on relative mRNA abundances of interleukin 6 in LPS-induced IPEC-J2 cells. **(B)** The effect of AKP on relative mRNA abundances of interleukin 8 in LPS-induced IPEC-J2 cells. **(C)** The effect of AKP on relative mRNA abundances of tumor necrosis factor α in LPS-induced IPEC-J2 cells. **(D)** The effect of AKP on relative mRNA abundances of ASC amino acid transporter 2 in LPS-induced IPEC-J2 cells. **(E)** The effect of AKP on relative mRNA abundances of zonula occludens protein-1 in LPS-induced IPEC-J2 cells. **(F)** The effect of AKP on relative mRNA abundances of occludin-3 in LPS-induced IPEC-J2 cells. Error bars, SD; *n* = 10. **Significant (*p* < 0.05), ***very significant (*p* < 0.01).

The effect of AKP on cytokine gene expression in IPEC-J2 cells is shown in Figures 4A–C. Compared with the control, there was significant effect on the relative gene expression of IL-6, IL-8, and TNF-α in the LPS treatment (*p* < 0.01). The DelSig*phoA* treatment has no significant effect on IL-6 and IL-8, and downregulates the mRNA abundance of TNF-α (*p* < 0.05). Nevertheless, the DelSigD_153_G-D_330_N treatment extremely significantly downregulated the gene expression of IL-6, IL-8, and TNF-α (*p* < 0.01). Compared with the control + LPS, the DelSig*phoA* + LPS and DelSigD_153_G-D_330_N + LPS treatments were extraordinarily significantly downregulated on the mRNA abundance of IL-6, IL-8, and TNF-α (*p* < 0.01).

The effect of AKP on transporter gene expression in IPEC-J2 cells is shown in Figure 4D. Compared with the control, LPS treatment was significantly upregulated on the relative gene expression of ASCT-2 (*p* < 0.01). Nevertheless, the DelSig*phoA* and DelSigD_153_G-D_330_N treatments significantly downregulated the gene expression of ASCT-2 (*p* < 0.01). Compared with the control + LPS, the DelSig*phoA* + LPS and DelSigD_153_G-D_330_N + LPS treatments were extraordinarily significantly downregulated on ASCT-2 (*p* < 0.01).

The effect of AKP on gene expression of tight junction proteins in IPEC-J2 cells (Figures 4E,F). Compared with the control, LPS significantly downregulated the expression of ZO-1 and CLDN-3 (*p* < 0.01). Nevertheless, the DelSig*phoA* and DelSigD_153_G-D_330_N treatment groups significantly upregulated the expression of ZO-1 and CLDN-3 (*p* < 0.01). Compared with the control + LPS, the DelSig*phoA* + LPS and DelSigD_153_G-D_330_N + LPS treatment groups significantly upregulated the gene expression of ZO-1 and CLDN-3 (*p* < 0.01).

## Discussion

Weaned piglets are prone to bacterial flora imbalance, excessive growth of harmful bacteria, and cause piglet diarrhea for changes in food status, nutritional composition, and physiological status. The additives could promote intestinal development, enhance immunity, increase production performance, and reduce the incidence of diarrhea in weaned pigs. Reasonable use of exogenous additives has important production significance for preventing and treating diarrhea.

Previous studies have shown that AKP activity of the single mutants D_330_N or D_153_G was three or five times higher than that of the wild-type *phoA* with the signal peptide in *E. coli* (Dealwis et al., 1995: 865-71, Le Du et al., 2002: 941-53). The activity of the double mutants D_153_G-D_330_N was 40 times higher than that of the wild-type *phoA* with the signal peptide in *E. coli* (Le Du et al., 2002: 941-53). Our study found that the activity of DelSigD_153_G-D_330_N in *B. subtilis* was up to 32 times more than the DelSig*phoA*, and nearly 1,600 times more than that of wild-type *phoA* with the signal peptide (Figure 1). In our study, the signal peptide was deleted, and *B. subtilis* was used as the expression host. Aggregation phenomenon and low expression level of the protein occur due to the presence of signal peptide, and transmembrane segments have been highlighted by a number of researchers (Masomian et al., 2017: 51-62). In addition, *B. subtilis* easily absorbs extracellular genetic material for horizontal gene transfer as a nutrient, and it also shows super-strong extracellular secretion ability of proteins and metabolites, and without producing endotoxins (Chen et al., 2005: 1456-60).

In order to enhance the stability of the enzyme and maintain its enzyme activity, various technologies have been used to form capsules including spray drying, spray cooling, extrusion coating, fluidized bed coating, liposome retention, coagulation, inclusion compounding, centrifugal extrusion, and rotating suspension separation (Gibbs et al., 1999: 213-24). In the spray drying of food, the hot air usually maintains 0.8∼1.0 s. In our study, the DelSig*phoA* and DelSigD_153_G-D_330_N still maintained high activity after being treated at 120°C for 1 min in Figure 3B.

It is well known that the increase in pro-inflammatory factors leads to the destruction and localization of tight junction protein levels, increasing the passage of intestinal contents into the systemic circulation. Therefore, proinflammatory cytokine expression levels were widely considered to be an effective target for the treatment of inflammation (Liu et al., 2016: 1009-17). LPS triggers the inflammatory cascade by binding to TLR-4 on the apical membrane of PTEC, leading to the release in a variety of inflammatory mediators, including ATP and ADP, which further enhance the proinflammatory cascade through purine P2 receptor signaling (Peters et al., 2015: 4932-45). First of all, AKP dephosphorylates LPS for detoxification, thereby preventing TLR-4 activation and reducing the production of proinflammatory factors (Liu et al., 2016: 1009-17). In addition, AKP dephosphorylates ATP and ADP to adenosine, thereby providing protection by reducing P2 receptor activation. This explains the reduction of proinflammatory cytokines when AKP alone was applied and the better performance of AKP in alleviating cellular inflammation under LPS stimulation, in correspondence with the previous study (Peters et al., 2015: 4932-45). Moreover, the adenosine activates AMPK through specific inhibitors to promote the maintenance of the integrity of the junction complex (Galardo et al., 2010: 49-58). However, there is no explanation, at present, why the tight junction protein expression is increased in cells treated with AKP and LPS than in cells treated with AKP alone. The adenosine is probably responsible for this phenomenon, but we need to further confirm it in the future. ASCT-2 is a key regulator of glutamine uptake in CD4 + T cells and influences the development of proinflammatory Th1 and Th17 responses *in vivo* and *in vitro* (Poffenberger and Jones, 2014: 635-7). AKP prevents the occurrence of proinflammatory reactions by downregulating the gene expression of ASCT2, consistent with the previous study (Xu et al., 2020: 111376). These results postulate that LPS exposure causes intestinal inflammation and apoptosis, thereby disrupting intestinal barrier function and affecting absorption and transport. AKP preconditioning may prevent LPS damage by regulating the mRNA abundance of transporter genes to some extent (Figure 4).

## Data Availability Statement

The original contributions presented in the study are included in the article/Supplementary Material, further inquiries can be directed to the corresponding author/s.

## Author Contributions

JiaY, HY, and JL contributed to the conceiving and designing of the experiments. YD and YX wrote the original draft of the manuscript. YD, JieY, YS, SY, QL, LW, DZ, YZ, CC, QH, and YW performed the cell-treating trial and sample collection. YD conducted the strain screening and alkaline phosphatase assay. YD, YW, CW, and YZ contributed to the protein structure simulation analysis experiment. JiaY, YX, MA, HY, and JL reviewed and revised the manuscript. JiaY obtained the funding and supervised the project. All authors have contributed to the article and approved the submitted version.

## Conflict of Interest

The authors declare that the research was conducted in the absence of any commercial or financial relationships that could be construed as a potential conflict of interest.

## Publisher’s Note

All claims expressed in this article are solely those of the authors and do not necessarily represent those of their affiliated organizations, or those of the publisher, the editors and the reviewers. Any product that may be evaluated in this article, or claim that may be made by its manufacturer, is not guaranteed or endorsed by the publisher.

## Footnotes

1
http://www.otwobiotech.com/

2
http://www.hzsjq.com/

3
https://www.ncbi.nlm.nih.gov/

4
www.cbs.dtu.dk/services/SignalP/

5
https://mail.sangon.com/

6
http://www.njjcbio.com/

## Supplementary Material

Supplementary Figure 1 The three initial structures ligand interaction diagram. Panel **(A)** is the binding pocket of AKP-ligand complex; panel **(B)** is the binding pocket of DelSig*phoA*-ligand complex; panel **(C)** is the binding pocket of DelSigD_153_G-D_330_N-ligand complex. The yellow dash lines represent the hydrogen bonds, magenta dash lines represent salt bridges, cyan dash lines represent the Pi- Pi stacking interactions, green dash lines represent Pi-cation interactions.

Supplementary Figure 2 Plasmid stability of D_153_G-D_330_N in *B. subtilis*. The culture was transferred into the new LB medium, and the number of cells was counted on plates with and without kanamycin (resistance colony) in each generation after culturing for 12 h. The ratio of kanamycin resistant clones to total cells was calculated. Error bars, SD; *n* = 3.
