# Supplementary material for: Optimization, Characteristics, and Functions of Alkaline Phosphatase From Escherichia coli
Source: Front Microbiol. 2022 Feb 21;12:761189. doi: 10.3389/fmicb.2021.761189 (PMC8899610; doi:10.3389/fmicb.2021.761189)
Supplement: Supplementary file 1 [file Table_1.docx]

Table S1. *E. coli* strains and plasmids used in the study.

| Strain/plasmid | Description | Features | Ref. |
| --- | --- | --- | --- |
| **Strain** |  |  |  |
| **GB05-dir** | GB2005, araC-BAD-ETγA (*E. coli*) | Plasmid construction | (Fu, et al., 2012: 440-6) |
| ***B. subtilis* 168** | Wild type strain | Expression host | (Cui, et al., 2018: 70) |
| **Plasmid** |  |  |  |
| **pGB-Cm-ccdA-Ptet-*phoA*** | *phoA* from *E. coli* | pGB,*phoA*,Cm^r^ | This study |
| **pGB-Cm-ccdA-Ptet-*phoE*** | *phoE* from *B. subtilis* | pGB,*phoE*,Cm^r^ | This study |
| **pGB-Cm-ccdA-Ptet-*nudF*** | *nudF* from *B. subtilis* | pGB,*nudF*,Cm^r^ | This study |
| **pGB-Cm-ccdA-Ptet-*apn*** | *apn* from pig | pGB,*apn*,Cm^r^ | This study |
| **pP43NMK-P43-Km** | Empty vector | pP43NMK, Km^r^ | This study |
| **pP43NMK-P43-p*hoA*-Km** | *phoA* from *E. coli* | pP43NMK, *PhoA*, Km^r^ | This study |
| **pP43NMK-P43-*phoE*-Km** | *phoE* from *B. subtilis* | pP43NMK, *PhoE*,Km^r^ | This study |
| **pP43NMK-P43-*nucF*-Km** | *nudF* from *B. subtilis* | pP43NMK, *NucF*, Km^r^ | This study |
| **pP43NMK-P43-*apn*-Cm-Km** | *apn* from pig | pP43NMK, Cm^r^,Km^r^ | This study |
| **pP43NMK-P43-DelSig*phoA*-Km** | *phoA* without signal peptide | pP43NMK, *phoA*,Km^r^ | This study |
| **pP43NMK-P43-DelSig*phoA*-D_101_A-E_322_Y-K_328_Y-D_153_G-D_330_N** | D_101_A-E_322_Y-K_328_Y-D_153_G-D_330_N mutaion | pP43NMK, *phoA*,Km^r^ | This study |
| **pP43NMK-P43-DelSig*phoA*-E_322_Y-K_328_Y-D_153_G-D_330_N** | E_322_Y-K_328_Y-D_153_G-D_330_N mutaion | pP43NMK, *phoA*,Km^r^ | This study |
| **pP43NMK-P43-DelSig*phoA*-D_101_A-E_322_Y-K_328_Y** | D_101_A-E_322_Y-K_328_Y mutaion | pP43NMK, *phoA*, Km^r^ | This study |
| **pP43NMK-P43-DelSig*phoA*-D_101_A-D_153_G-D_330_N** | D_101_A-D_153_G-D_330_N mutaion | pP43NMK, *phoA*,Km^r^ | This study |
| **pP43NMK-P43-DelSig*phoA*-D_101_A** | D_101_A mutaion | pP43NMK, *phoA*,Km^r^ | This study |
| **pP43NMK-P43-DelSig*phoA*-E_322_Y-K_328_Y** | E_322_Y-K_328_Y mutaion | pP43NMK, *phoA*,Km^r^ | This study |
| **pP43NMK-P43-DelSig*phoA*-D_153_G-D_330_N** | D_153_G-D_330_N mutaion | pP43NMK, *phoA*,Km^r^ | (Le Du, et al., 2002: 941-53) |
| **pP43NMK-P43-DelSig*phoA*-D_153_G-D_330_N-Km-Cm- mazEF** | D_153_G-D_330_N with toxin-antitoxin system mazEF | pP43NMK, *phoA*,Km^r^,Cm^r^ | This study |
