# Supplementary material for: Optimization, Characteristics, and Functions of Alkaline Phosphatase From Escherichia coli
Source: Front Microbiol. 2022 Feb 21;12:761189. doi: 10.3389/fmicb.2021.761189 (PMC8899610; doi:10.3389/fmicb.2021.761189)
Supplement: Supplementary file 2 [file Table_2.docx]

Table S2. Configuration materials for medium GM Ⅰ and GM Ⅱ

| Composition | GM I | GM II |
| --- | --- | --- |
| K_2_HPO_4_ | 13.4 g/L | 13.4 g/L |
| KH_2_PO_4_ | 5.7 g/L | 5.7 g/L |
| （NH_4_）_2_SO_4_ | 1.9 g/L | 1.9 g/L |
| Na_3_C_6_H_5_O_7_·2H_2_O | 1 g/L | 1 g/L |
| MgSO_4_.7H_2_O | 0.2 g/L | 0.2 g/L |
| glucose | 0.5 g/L | 0.5 g/L |
| Casein | 0.2 g/L | 0.04 g/L |
| Yeast extract | 1 g/L | 0.04 g/L |
| MgCl_2_ | / | 0.51 g/L |
| CaCl_2_ | / | 0.37 g/L |
