# Supplementary material for: Optimization, Characteristics, and Functions of Alkaline Phosphatase From Escherichia coli
Source: Front Microbiol. 2022 Feb 21;12:761189. doi: 10.3389/fmicb.2021.761189 (PMC8899610; doi:10.3389/fmicb.2021.761189)
Supplement: Supplementary file 3 [file Table_3.docx]

Table S3. Oligonucleotide sequences used in the study

| **Name** | **Sequence (5’-3’)** | **Application** |  |
| --- | --- | --- | --- |
| **P43-PhoA-5** | cggcccaaatggtttttgtaacacatgcctcagctgcaaaacaaagcactattgcactg | Amplify the cloning *phoA* |  |
| **P43-PhoA-3** | GTTGTGTGGAATTGTGAGCGatatcAAGTCCAGCTGCAGttatttcagccccagagcgg |  |  |
| **P43-PhoE-5** | cggcccaaatggtttttgtaacacatgcctcagctgcaacagccgtttgtttagtaaga | Amplify the cloning *phoE* |  |
| **P43-PhoE-3** | GTTGTGTGGAATTGTGAGCGatatcAAGTCCAGCTGCAGttatttgataaagccggata |  |  |
| **P43-NudF-5** | cggcccaaatggtttttgtaacacatgcctcagctgcaaaatcattagaagaaaaaaca | Amplify the cloning *nudF* |  |
| **P43-NudF-3**  **P43-DelSig*phoA* -5** | GTTGTGTGGAATTGTGAGCGatatcAAGTCCAGCTGCAGtcatttttgtgcttggagcg  cggcccaaatggtttttgtaacacatgcctcagctgcacggacaccagaaatgcctgtt |  |  |
|  |  | Amplify the cloning DelSig*phoA* |  |
| **DelSig*PhoA*-D153G-D330N** | |  |  |
| **A DelSigp*hoA* -5** | cggcccaaatggtttttgtaacacatgcctcagctgcacggacaccagaaatgcctgtt |  | |
| **A DelSig*phoA* -3** | ctgcaactctgcggtagaaac | Amplify the cloning DelSig*PhoA* -D_153_G-D_330_N |  |
| **B DelSigp*hoA* -5** | gtttctaccgcagagttgcagggcgccacgcccgctgcgctggtg |  |  |
| **B DelSig*phoA* -3** | cccacaaggattcgcagcatgattctgtttatcgattgacgcac |  |  |
| **C DelSig*phoA* -5** | catgctgcgaatccttgtggg |  |  |
| **C DelSig*phoA* -3** | GTTGTGTGGAATTGTGAGCGatatcAAGTCCAGCTGCAGttatttcagccccagagcgg |  |  |
| **Plasmid stabilization system** |  | Amplify the cloning maZEF stabilization system |  |
| **Cm-5** | TCTAAAATTATCTGAAAAGGGAATGAGAATAgatCCTGTCAAAGAACCATCAAACCCTT |  |  |
| **Cm-3** | TTATAAAAGCCAGTCATTAGGCCTATCTGACAATTC |  |  |
| **maZEF-5** | CCTAATGACTGGCTTTTATAAaggaggaagcttcATGATCCACAGTAGCGTAAAGCGT |  |  |
| **maZEF-3** | GCGTTTTGACACATCCACTATATATCCGTGTcgactacccaatcagtacgttaattttg |  |  |
| **qPCR** |  | (Orsolya, et al., 2016: e0166642) |  |
| **IL-6-5** | TTCACCTCTCCGGACAAAAC |  |  |
| **IL-6-3** | TCTGCCAGTACCTCCTTGCT |  |  |
| **IL-8-5** | AGAGGTCTGCCTGGACCCCA |  |  |
| **IL-8-3** | GGGAGCCACGGAGAATGGGT |  |  |
| **TNF-α-5** | TTCCAGCTGGCCCCTTGAGC |  |  |
| **TNF-α-3** | GAGGGCATTGGCATACCCAC |  |  |
| **ASCT2-5** | GCCAGCAAGATTGTGGAGAT | (Omonijo, et al., 2019: 615-24) |  |
| **ASCT2-3** | GAGCTGGATGAGGTTCCAAA |  |  |
| **CLDN3-5** | CTACGACCGCAAGGACTACG |  |  |
| **CLDN3-3** | TAGCATCTGGGTGGACTGGT |  |  |
| **ZO1-5** | GATCCTGACCCGGTGTCTGA | (Orsolya, et al., 2016: e0166642) |  |
| **ZO1-3** | TTGGTGGGTTTGGTGGGTT |  |  |
| **β actin- 5** | AGTTGAAGGTGGTCTCGTGG |  |  |
| **β actin- 3** | TGCGGGACATCAAGGAGAAG |  |  |
| **CycA-5** | GCGTCTCCTTCGAGCTGTT |  |  |
| **CycA-3** | CCATTATGGCGTGTGAAGTC |  |  |

Note: IL-6: Interleukin 6; IL-8: Interleukin 8; TNF-α: Tumor necrosis factor α; ASCT2: ASC amino acid transporter 2; CLDN3: Claudin 3; CycA: Cyclin A; ZO-1: zonula occludens protein-1.
