# Supplementary figures and images for: Optimization, Characteristics, and Functions of Alkaline Phosphatase From Escherichia coli
Source: Front Microbiol. 2022 Feb 21;12:761189. doi: 10.3389/fmicb.2021.761189 (PMC8899610; doi:10.3389/fmicb.2021.761189)

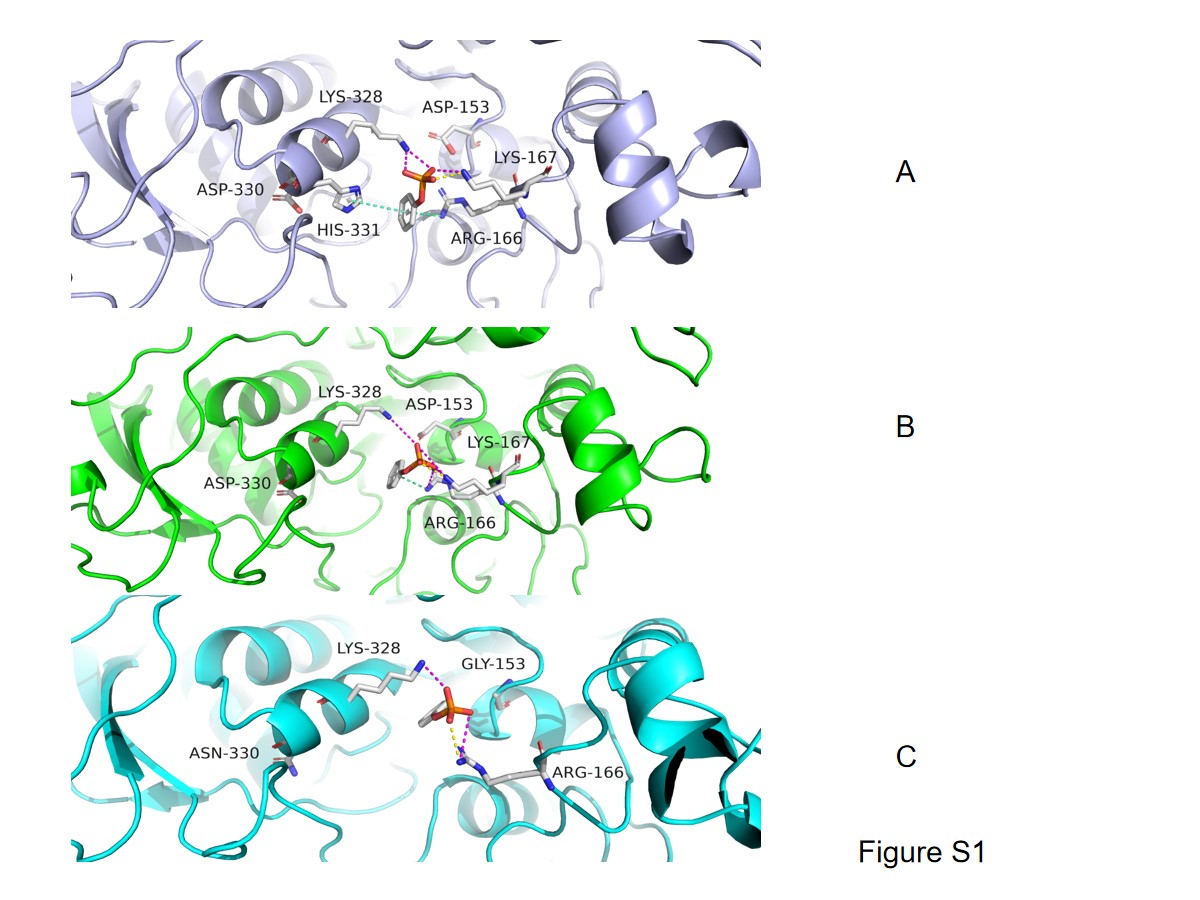

Supplement: Supplementary Figure 1 — The three initial structures ligand interaction diagram. Panel (A) is the binding pocket of AKP-ligand complex; panel (B) is the binding pocket of DelSigphoA-ligand complex; panel (C) is the binding pocket of DelSigD153G-D330N-ligand complex. The yellow dash lines represent the hydrogen bonds, magenta dash lines represent salt bridges, cyan dash lines represent the Pi- Pi stacking interactions, green dash lines represent Pi-cation interactions. [file Image_1.jpg]

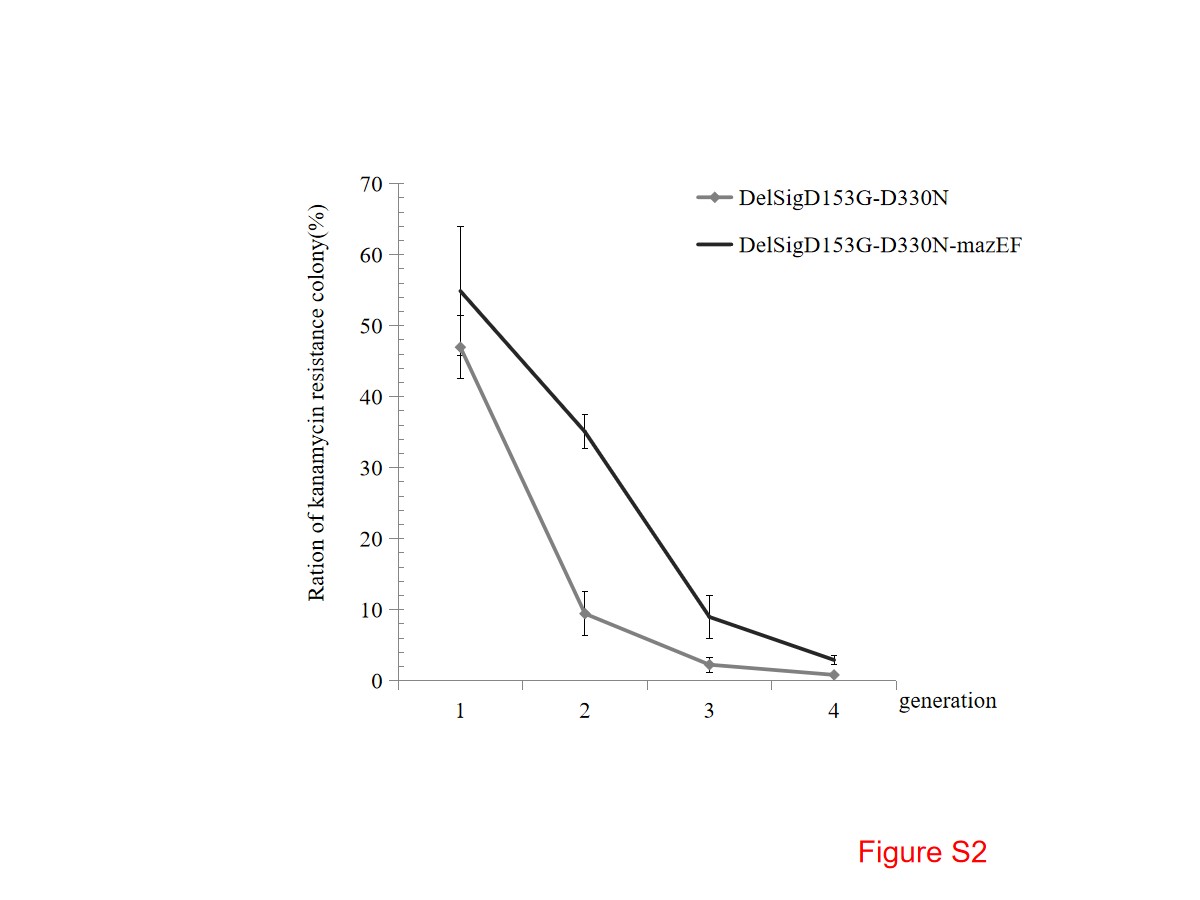

Supplement: Supplementary Figure 2 — Plasmid stability of D153G-D330N in B. subtilis. The culture was transferred into the new LB medium, and the number of cells was counted on plates with and without kanamycin (resistance colony) in each generation after culturing for 12 h. The ratio of kanamycin resistant clones to total cells was calculated. Error bars, SD; n = 3. [file Image_2.jpg]
